# Assessment of KN95 Mask Filtering Degradation and Breathing Detection: A Pilot Study

**DOI:** 10.3390/s25247623

**Published:** 2025-12-16

**Authors:** Julie Payette, Alexandre Perrotton, Paul Fourmont, Fabrice Vaussenat, Jaime A. Benavides, Luis Felipe Gerlein, Sylvain G. Cloutier

**Affiliations:** 1 Department of Electrical Engineering, Ecole de Technologie Superieure, Montreal, QC H3C 1K3, Canada; julie.payette.1@ens.etsmtl.ca (J.P.); paul.fourmont.1@etsmtl.net (P.F.); fabrice.vaussenat@lacime.etsmtl.ca (F.V.);; 2 Department of Materials Science & Engineering, Massachussetts Institute of Technology, Cambridge, MA 02139, USA

**Keywords:** humidity sensor array, KN95 mask, feature extraction, time-series analysis, occupational health, breathing monitoring

## Abstract

This study aims to monitor mask performance in operando using all-printed humidity sensor arrays based on BiFeO_3_/BiOCl heterostructures. Two screen-printed 19-sensor arrays are fixed directly atop the mask, in order to analyze moisture levels in exhaled breath and extract performance indicators. This approach allows for an examination of the humidity saturation and absorption over time during operation. Accumulation of moisture within the mask can affect its performance, and factors like breath humidity, mask material, and ambient conditions influence this. Results show that the measured data follows an exponential decay, achieving correlation factors of over 0.9 for all tests. We also detect breathing differences through feature extraction, investigating the respiration rates and signal amplitudes for both normal and deep breathing. Furthermore, we animated the airflow in the mask in both 2D and 3D, allowing for the eventual detection of leaks for ill-fitting masks. This study introduces an innovative approach for the assessment of mask fit and longevity, contributing to improving mask efficacy and public health outcomes.

## 1. Introduction

Respiratory disorders present a significant global health concern, with an estimated 544 million people suffering from chronic respiratory diseases in 2016 [1]. The global SARS-CoV-2 (COVID-19) pandemic also underscored the critical importance of respiratory health during one of the most challenging health crises in recent history [2].

Respiratory protection using N95/KN95 masks helped reduce transmission, providing a physical barrier against the inhalation of dust, bacteria, and airborne virus particles [3,4]. KN95 masks are designed to filter at least 95% of airborne particles of sizes down to 0.3 microns and are recognized as an effective tool to limit the spread of respiratory diseases like COVID-19 [5]. Still, incorrect positioning can significantly hamper their protection efficiency [6]. Studies show that improperly positioned masks significantly reduce protection effectiveness against airborne particles [7,8].

The qualification of KN95 facemasks involves several key parameters like filtration efficiency, fit testing, and breathability. Researchers have recently attempted to monitor mask performances in operando in order to achieve better protection and improve fundamental understandings [5]. The most promising approaches must still contend with factors like variability in breathing patterns and mask fitting among different users [9].

Printed sensor technologies provide a straightforward approach for mass production, which lowers costs and facilitates their integration into useful devices [10]. These arrays can potentially (1) provide more accurate and complete datasets compared to single sensor studies and (2) capture information about the local turbulence of airflow patterns inside the mask, thereby serving as an indicator of proper mask positioning [11]. This innovative approach could prove highly beneficial, favoring optimal efficacy and, ultimately, public health.

### Related Work

Recent years have seen a push to integrate wearable sensors and electronics into face masks to make them more efficient, reliable, and versatile [12,13,14]. These smart masks hold great potential, enabling the capture of complex physical, chemical, environmental, and biological information and patterns [13]. Depending on the intended application, various nanomaterials are employed, such as carbon aerogels [15], MXenes [16], or Au-TiO_2_ nanocomposites [17].

Some recent studies have explored single-sensor systems for breathing detection and monitoring. Ultrathin pressure sensors inserted into face masks were used for breathing detection [18]. Temperature-sensitive LSMO/Mica sensors placed under the nose were also used to monitor various breathing patterns [19]. A paper-based pressure sensor configuration was also used to distinguish between normal, deep, and held breaths based on electrical current variations [20].

While these single-sensor studies yielded some success, they remain limited to a specific area on the mask. For this reason, other studies explored sensor arrays as a way to add spatial information into the datasets. Graphene-based coatings used as strain sensors were applied on surgical masks, detecting breathing by measuring strain-induced resistance changes [21]. A 4 × 8 array of similar pressure sensors was arranged from nose to chin along the inner mask’s surface to achieve 2D respiration profiling [22]. While these studies demonstrate the potential of sensor arrays, their primary goal remains material characterization and development.

In this study, we focus instead on their improved ability to shed light on complex breathing patterns and their use for mask degradation assessments. BiFeO_3_ offers unique biocompatibility properties, making it a well-suited material system for humidity sensing. Indeed, recent advances in BiFeO_3_ sensing materials highlight several complementary strategies for enhancing humidity detection. Surface-area engineering is one approach, as demonstrated by BFO nanoparticles combined with carbon fibers, which create highly porous composites that increase the number of active adsorption sites [23]. Defect and ferroelectric engineering is another approach. The introduction of oxygen vacancies, together with controlled ferroelectric polarization, produces strong internal electric fields and efficient charge-transport pathways, resulting in highly sensitive and rapid humidity responses [24]. BFO nanofibers obtained by electrospinning further contribute to improved sensing through their large surface-to-volume ratio and mechanical robustness [25]. More recently, printed BFO-based heterojunctions, particularly BiFeO_3_/BiOCl systems, have shown that combining BFO with a suitable partner semiconductor can extend the operational humidity range and increase signal stability [26].

In this work, a printed array of such sensors is used to investigate the complex spatial patterns and temporal variations in the airflow and filtering capacity of a commercial KN95 mask. For researchers and designers, this can provide a new and unique window to understand complex structure–property interrelations and ultimately improve their performance, lifetime, and efficiency.

Using this printed array of BFO-BiOCl heterostructures-based humidity sensors we then capture detailed breathing patterns, tracking variations in airflow. We successfully monitor humidity levels over time and across the mask surface. This innovative approach provides insights into mask fit and highlights markers for optimal mask lifespan and usage.

Most importantly, this work provides insights into long-term mask performance by evaluating humidity accumulation and absorption, which affects both breathability and filtration efficiency [27]. Given that factors such as breath humidity, mask materials, and environmental conditions influence these properties, further study is essential to fully understand and enhance mask effectiveness [28].

## 2. Materials and Methods

### 2.1. Fabrication of Printed Humidity Sensors

Humidity sensors are screen-printed using BiFeO_3_ powder dispersed in a commercial ink vehicle provided by Henkel (Düsseldorf, Germany) (SOL725). The fabrication steps for the BiFeO_3_ powders and humidity sensors have already been described in our previous studies [10,29]. As reported, both the amount of BiFeO_3_ powder and the annealing temperature are be optimized to obtain the best sensing performances. Tuning such parameters allows us to reach the highest surface area and increase the sensitivity of the sensors, which is an important parameter for any humidity sensors. From previous studies, we established that BiOCl nanosheets grow atop BiFeO_3_ particles due to chemical etching. This chemical reaction takes place during evaporation of the ink vehicle when annealing the sample to 300 °C [10]. Such heterostructures increase the performances of the printed sensors, as a seven-fold increase in the active surface area has been reported. Due to the sensing material, the sensor’s behavior is reversed in relation to humidity, i.e., resistance decreases as humidity increases. Characterization of this material was carried out in other studies by the co-author Paul Fourmont, where XRD, thickness, and calibration data can be found [10,26].

**Figure 1:**
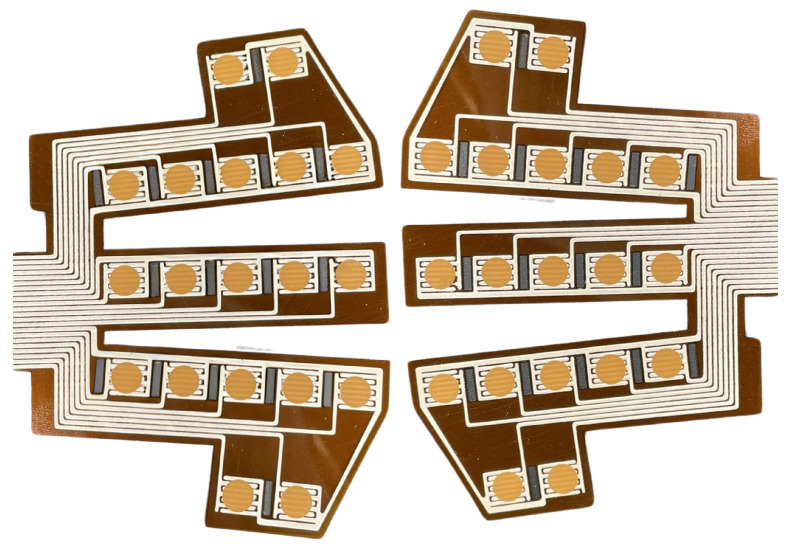
BFO humidity sensor arrays designed to fit masks. Orange dots are the active layer, printed atop the conductive silver interdigitated electrodes.

The arrays of humidity sensors are printed using a methodology that involves printing two successive layers on a polyimide sheet. Firstly, an EDAG725 silver ink (Henkel, Düsseldorf, Germany) [30] layer is screen-printed to form conductive silver interdigitated electrodes. This layer is then baked at 300 °C for an hour to remove the solvent and ensure an optimal electrical conductivity. Secondly, the active layer made of SOL725 and BiFeO_3_ powder is screen-printed atop the silver traces. Polyimide sheets are then annealed at 300 °C for 10 min to enable the growth of BiOCl nanosheets atop the BiFeO_3_ particles, for a final thickness of 14 ± 2 µm. As shown in Figure 1, arrays made of 19 sensors are printed on each polyimide sheet to collect relevant data.

### 2.2. Experimental Setup

**Figure 2:**
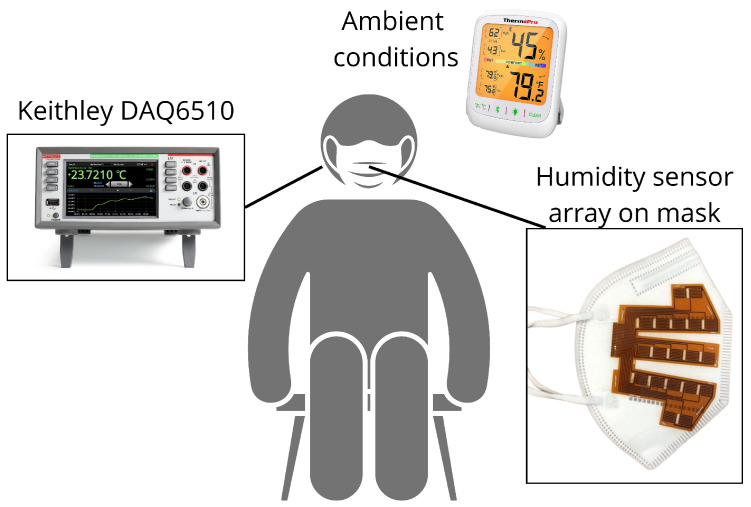
Schematic representation of our experimental configuration.

After printing humidity sensor arrays, polyimide sheets are laser cut using an LPFK Protolaser U3 (LPFK, Garbsen, Germany). Two polyimide sheets are then symmetrically sewn on each side of a KN95-type mask with the sensors facing it, as shown in Figure 2. Both sensor arrays are connected to a Keithley DAQ6510 data logger (Tektronix, Beaverton, OR, USA) [31] to ensure an optimal reading of the electrical resistance values from each sensor. This device enables the measurement and data collection of all electrical resistance variations via multiplexed sampling with 3 Hz frequency. To ensure that no screen-printed sensor is defective, an evaluation phase is first performed. The electrical resistance of all the sensors is measured after being attached to the KN95 mask, before it is worn. The measurements taken outside the mask offer an indication of relative humidity permeation, thus providing a depiction of moisture accumulation within the mask. Therefore, all the electrical resistance values are related to the relative humidity of the room [32]. The ambient temperature and humidity are recorded throughout the entire data collection with a ThermoPro TP359 (ThermoPro, Toronto, ON, Canada) which has tolerances of ±0.2 °C and ±2% RH.

### 2.3. Experimental Protocol

For this test, we recruited four healthy participants through convenience sampling. All participants (two women, two men, age 33.0 ± 12.1 years) gave informed consent before participating in the study, which was approved by the institutional Ethics Committee (Certificate H20240806). Participants had to be able to wear the KN95 mask for 35 min and be non-smokers. During the measurements, the participants were seated on stable chairs with armrests to ensure stable data collection [33]. The measurements were repeated on three consecutive days. Each of the four participants wore their assigned mask on all tests, keeping the same sensors throughout. The experimental configuration is illustrated in Figure 2.

Following the initial compliance assessment of all sensors, we initiated a two min data recording of the electrical resistance of all the sensors without wearing the KN95 mask. This step allowed us to assess the relative humidity of the room, which served as a baseline for the calibration of the mask. Following this, the subjects were instructed to wear the mask. All participants adhered to the subsequent protocol: normal respiration for four minutes followed by deep breathing for one minute—both inhales and exhales though the nose—and this was repeated six times for all participants, for a total duration of 30 min [9]. The resistance was then measured for another two min, without wearing the mask, at the end of the protocol. This step was added to verify how the humidity in the mask changes immediately after it is removed.

### 2.4. Kinetic Modeling

From the electrical resistances values obtained during our measurements, several parameters can be used in a kinetic model to analyze the variation in the data. We find that three parameters (a,b,c) can be used in a kinetic model to accurately model the mask’s behavior and the data variability. The kinetic model relies on a first-order equation linked to the diffusion of water particles through a membrane represented by Fick’s law, which states that the amount of gas that goes through a sheet of tissue is proportional to its area, but inversely proportional to its thickness [34].

(1) $$J=-D\frac{d\phi }{dx}$$

where *J* represents the diffusion flux, *D* is the diffusion coefficient, and $\frac{d\phi }{dx}$ is the concentration gradient [35].

Fick’s second law,

(2) $$\frac{\partial \phi }{\partial t}=D\frac{\partial^{2}\phi }{\partial x^{2}},$$

takes into account the temporal change in concentration due to diffusion. This equation reflects the physical constraints of the mask’s material and the inevitable slowdown of the absorption rate once the material reaches saturation [36].

Solving this equation for a membrane of finite thickness yields a series of exponentially decaying eigenmodes. Over longer durations, all higher-order modes decay rapidly, and the solution is dominated by the slowest mode, which is why the kinetics of the measured electrical resistances follow the equation of exponential decay:

(3) $$R\left(t\right)=ae^{-bt}+c+r\left(t\right)$$

The mathematical representation of this kinetic model relates to the fundamental principles found in Fick’s laws of diffusion. The term r(t) represents the variable volume of inhaled and exhaled respiratory air, directly influenced by variations in lung volume [37], but was not considered in this modeling.

In this equation, *c* represents the equilibrium resistance/humidity reached at t→∞ once the internal water concentration has equilibrated with the external humidity. The coefficient $a=R_{0}-c$ corresponds to the amplitude of the resistance change following a humidity step, and reflects the difference between the initial and final moisture contents within the membrane. The parameter *b* is the decay rate and equals the inverse of the characteristic time constant associated with moisture transport inside the membrane. Therefore, it indicates the speed at which moisture permeates through the mask, reflecting how swiftly molecules move from an area of higher concentration to an area of lower concentration [38].

### 2.5. Data Processing and Analysis

Signal processing and data analysis is performed using Python (version 3.12.4). First, motion-related outliers are removed from the rolling mean of the raw data. These erratic values are mostly generated when participants have to put on or take off the mask during the data collection. Not only are these values removed, but an interpolation of the signal is computed to replace them. Then, to detect the differences between normal and deep breathing patterns, we extract features from the signal for an easier and more telling detection than only the electrical resistance signal. Using SciPy’s library of functions (version 1.15.0), it is possible to detect the peaks in the signal, which in this case are the duration of each breath Δt. Once this feature is acquired, we can then compute other features for each breath. To ensure that this Δt is valid, an algorithm has also been implemented to identify anomalies in breathing duration. If a 1 s breath is measured, the neighboring data points are evaluated manually to determine whether the peak detection is erroneous. For this work, we extract the respiration rate $\left(RR=\frac{60}{\Delta t}\right)$, its frequency $\left(f=\frac{1}{\Delta t}\right)$, and the maximal and minimal resistance values in order to obtain the signal’s amplitude $\left(A=R_{max}-R_{min}\right)$. With this information, the detection of the breathing mode is more direct than only relying on the resistance, since its mean value remains similar, while the amplitude differs between deep and normal breathing.

**Figure 3:**
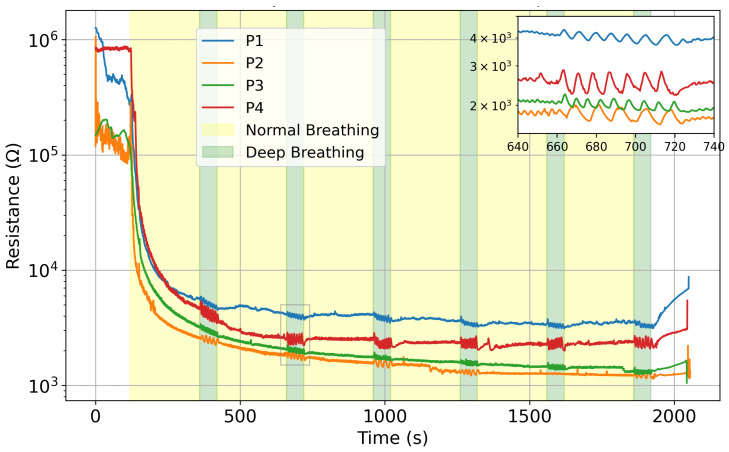
Mean resistance value comparison on day 2 for all four participants, in logarithmic scale. Zoomed-in portion shows the variation between normal and deep breathing.

For the curve fitting model, we use SciPy’s curve_fit function. Based upon the visual appearance of the resistance curve and the sensors’ behavior (Figure 3), it appears clear that the signal shows similarity to an exponential decay. Thus, it is the function from Equation (3) that is used to fit the curve. Due to the abrupt changes occurring in the signal before and after wearing the mask, we only fit the data during the stable breathing period (200 s < t < 1920 s).

## 3. Results and Discussion

### 3.1. Mask Filtering Assessment

Figure 3 plots the average electrical resistance of the sensors for all participants on day 2; the difference between normal and deep breathing is noticeable in all four cases. The humidity sensors show the same behavior with a different trend line, depending on each user. The difference in amplitude is highlighted in the inset in Figure 3. During some of the tests, certain sensors encountered issues, resulting in unreliable signals. This is due to the connector to the acquisition system, which can apply strain to the printed circuit and lose signal. Consequently, their misreadings were excluded from the calculation of the array’s mean signal to ensure accuracy (details are reported in Supplementary Information Section, Figure S1 and Table S1).

**Figure 4:**
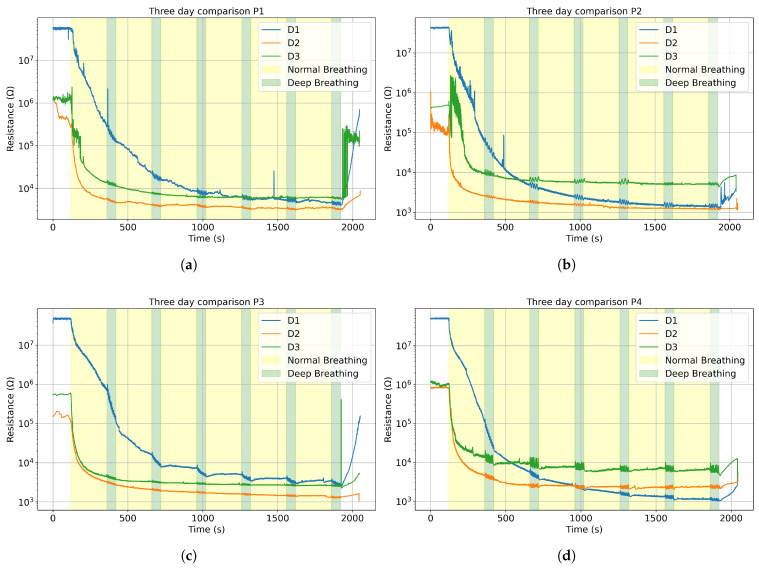
The average resistance curve for three consecutive days in logarithmic scale for (**a**) Participant 1; (**b**) Participant 2; (**c**) Participant 3; (**d**) Participant 4.

The mask membrane absorbs moisture as the users breathe. However, as the membrane dries between uses, its baselines slowly returns towards its initial state, due to a lower humidity in the room. Such features are determined by observing different initial values for consecutive days. Figure 4 shows the evolution using 24 h intervals. Indeed, the mask rids itself of most of its trapped humidity in the ambient conditions detailed in Table 1. The ambient humidity was lower on day 1, but similar for day 2 and 3 (around 50% RH). We can notice that sensors dry up. Altogether, the starting value and trend line depend on relative ambient humidity.

Since the sensors’ resistance changes inversely to humidity, a lower humidity will increase the initial electrical resistance value [29]. Therefore, on Figure 4 for each participant, we see the same similar initial electrical resistance values for the three consecutive days, consistent with the mean humidity values: day 1 (26.5%), day 2 (53.0%) and day 3 (51.0%). For day 1, since the relative humidity was so low, the data acquisition system fluctuated between values around $10^{8}$ and *“overflow”*, indicating that the values exceeded the maximum sensitivity range, so we limited those values at $10^{8}$, which explains why all the day 1 curves show similar starting points around $10^{8}$.

**Figure 5:**
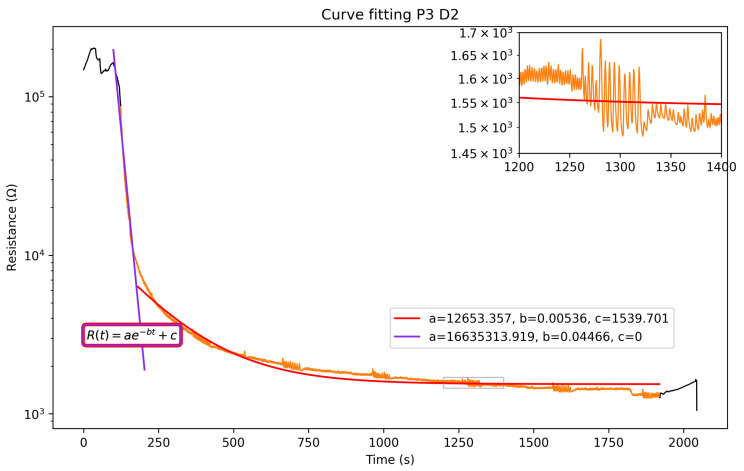
Fitting of the electrical resistance of the humidity sensors. Black segments are before/after breathing. Orange segment is duration of breathing.

The sensors’ signal can be divided into three main phases: before, during, and after each breathing session. The measured electrical resistance drops significantly once the mask is worn and rises up as soon as it is taken off, sometimes creating peaks and instability in the data, as seen in Figure 4a. During the breathing, the sensors stabilize around a baseline. As mentioned previously, the sensors’ response is modeled to fit an exponential decay function. Figure 5 shows the compliance between the function and the data for P3 on D2, with an $R^{2}=0.987$. Only one set of measurements is reported in Figure 5, but all data follow a similar trend and are reported in the Supplementary Materials (Figure S2).

**Figure 6:**
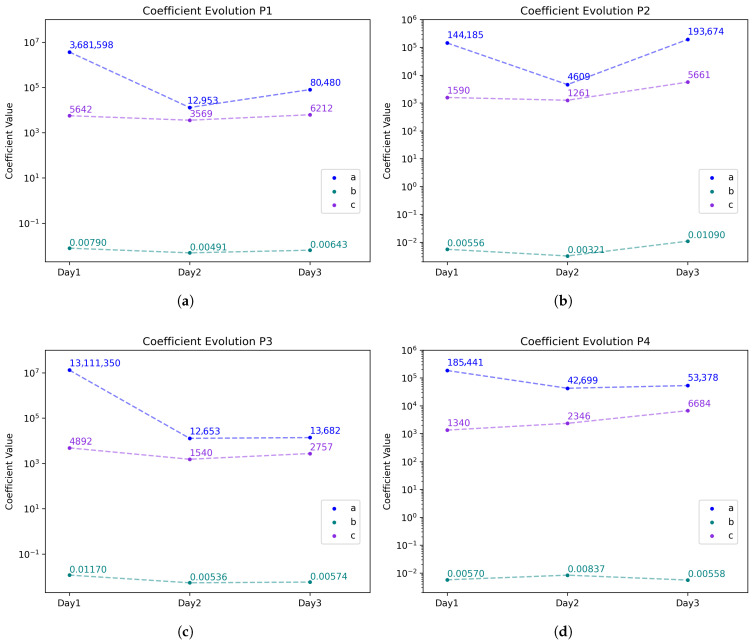
Coefficient comparison for all tests with the model equation $R\left(t\right)=\log\left(ae^{-bt}\right)+c$ for (**a**) Participant 1; (**b**) Participant 2; (**c**) Participant 3; (**d**) Participant 4. The corresponding curves can be found in the Supplementary Materials in Figure S2.

Table 2 includes computed $R^{2}$ coefficients for all participants and Figure 6 shows the variation in the model’s coefficients a,b,c from the exponential decay Equation (3). Although the decay constant *b* stays consistent throughout the tests, the filtering degradation remains similar, and the ambient conditions parameters *a* and *c* do change.

The model used to fit the data is an exponential $R\left(t\right)=ae^{-bt}+c$. A logarithmic scale is used for better readability, and the exponential’s coefficients change throughout the time interval of 0 to 2000 s. For small values of *t*,

(4) $$\log\left(ae^{-bt}\right)+c\approx \log\left(ae^{-bt}\right)=\log\left(a\right)-bt$$

the constant *c* will be negligible in size compared to the exponential, simplifying the equation into a straight line, where log(a) is the intercept and −b is the slope. However, as *t* increases, the exponential part of Equation (4) becomes negligible, resulting in log(c), which is the saturation baseline of our sensors. As the sensors’ humidity changes are fairly drastic, the purple line shows the initial descending slope, while the red line fits the saturation of the mask’s sensors. This second fitting enables the extraction of the interesting parameters related to decay and saturation. Coefficient *a* of the first fitting (in purple) gives a more accurate initial value than the second one would.

Although the decay constant *b* stays consistent throughout the tests, because the filtering degradation of the masks’ membranes remains similar, the ambient conditions parameters *a* (initial value) and *c* (saturation value) do change. The variation in the coefficients a,b, and *c*, used in Equation (4) to fit the data of all participants, is shown in Figure 6. Similar trends are found for all tests. However some differences are visible for P2 and P4 and are attributed to the relative humidity increase by 10% compared to P1 and P3 on Day 1 (Table 1).

### 3.2. Breathing Detection

**Figure 7:**
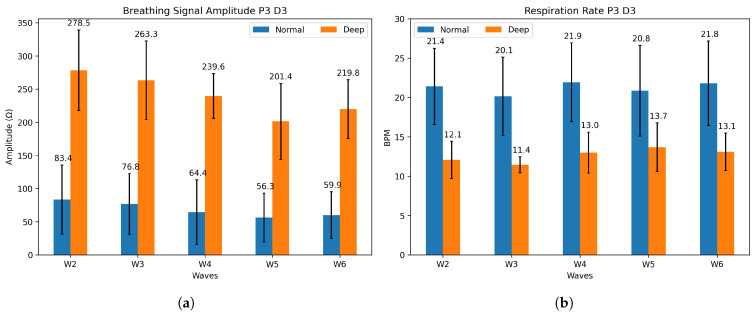
(**a**) Mean signal amplitude and (**b**) mean respiration rate for P3 on day 3, for both normal (blue) and deep (orange) breathing.

The integration of humidity sensor arrays with the masks enables the detection of different breathing patterns and air flow. As explained in Section 2, a feature extraction can be performed on the raw data to identify the breathing type (normal or deep). Figure 7a shows the distinction in signal amplitude between deep (average 240 Ω) and normal (average 68 Ω) breathing for Participant 3 on day 3. The five-minute cycles of breathing are referred to as waves. Wave 1 is not considered, as it takes place while the sensors are still absorbing humidity and not saturated. Figure 7b, on the other hand, displays the differences in respiration rate; with longer and deeper breaths, the respiration rate (RR) decreases. For Participant 3, an average variation of 7.7 breaths per minute between regular and deep breathing is measured. The respiration rate depends on the participant’s breathing, but a clear difference between both patterns is visible, as reported in Table 3. Wave 5 of deep breathing for Participant 3 on day 2 is not available, as the participant missed the cue. The normal breathing rate detected is within the medical standard, which states that it should be between 12 and 20 breaths per minute (bpm) [39]. The average signal amplitude difference is included in the table, but no repeatable trend or pattern is detected. Signal amplitude was inconclusive on day 1 for Participant 1 and is shown in the Supplementary Materials. Additional signal amplitude and respiration rate are shown in Figures S3–S6 for comparison.

**Figure 8:**
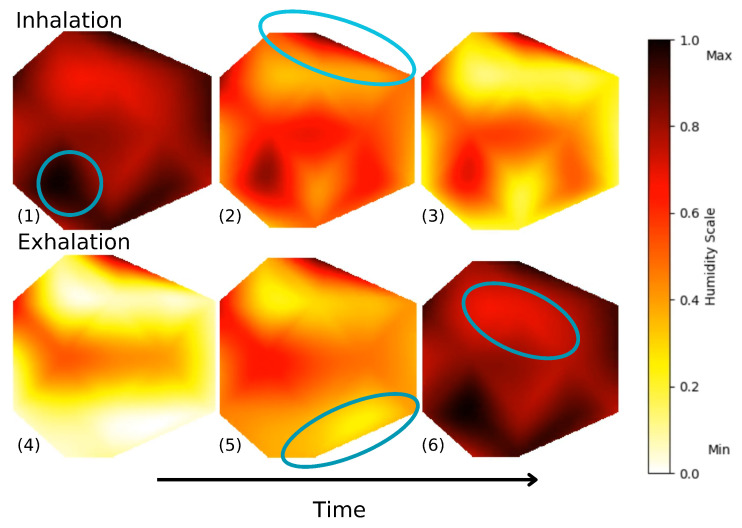
Frames of animated 2D air flow in left section of the mask during deep breathing. Video S2 in the Supplementary Materials shows both sides of the mask during a 2 min interval.

Using sensor arrays allows us to cover the entire mask surface, allowing for the detection of breathing patterns and also possible leakage attributed to a displacement or to improper fitting of the mask. Indeed, we are able to plot and visualize, both in 2D and 3D, the humidity movements during breathing. Figure 8 shows the variation in electrical resistance due to humidity variation for inhalation and exhalation. To map the signals, normalization of data on a scale from 0 to 1 is performed with the extrema $X^{\prime }=\frac{X-X_{min}}{X_{max}-X_{min}}$, where 0 indicates minimal humidity (after inhaling) and 1 indicates maximal humidity (after exhaling). During inhalation, cooler air goes through the mask. On the contrary, exhaled air is warmer and moisture-laden. This analysis can be separated into four different sections on the mask, identified in Figure 8. The sensors below the nose and in front of the mouth (1) show the most noticeable resistivity changes. They go from minimal to maximal humidity during a breath cycle. For the top sensors (2), the humidity stays at a minimal value, which indicates that the mask is well-fitted. The exhaled air does not escape through the top part of the face mask and external air does not come in through that part. This may also be due to the fact that these sensors are less impacted by breathing. The bottom sensors (5) show some changes in electrical resistance due to their positions on the face mask, below the nose, where exhaled air flows downward and outward. Furthermore, the KN95 masks have a malleable metal rod on the nose that ensures an optimal fit, whereas the part underneath the chin does not possess such a feature, resulting in a looser fit. The side sensors (6), located on the cheeks, show moderate changes, as they are mainly influenced by the fit of the mask and are less exposed to the airflow induced by the breath. Humidity does increase during exhaling, as some air disperses to the sides, but the normalized resistance stays quasi-constant in the upper range of our scale.

**Figure 9:**
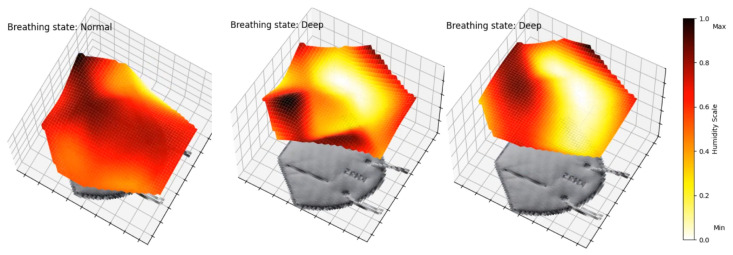
Frames of animated 3D air flow in left section of mask. Video S1 in Supplementary Materials of a 2 min interval.

We have also programmed an animation of the mask’s membrane in 3D. Figure 9 shows frames of Video S1, available in the Supplementary Materials. This animation enables a complete perception and visualization of the airflow and detects irregularities. In future work, this type of representation could be potentially useful to allow for better comparisons between experimental data and computational fluid dynamics model predictions.

### 3.3. Limitations and Future Work

This study aimed to detect different breathing patterns and model the behavior of the sensors stitched on the mask. However, this approach also presents some limitations. For this pilot study, the collected data suggest that breathing patterns can be successfully detected through different breathing phases. This concept will certainly need to be expanded in future tests using more extensive datasets and a more diverse participant pool. We also assumed that the humidity sensors are consistent with a reference tool, as has been demonstrated in one of our previous studies [40], with an error rate of 0.03 breaths/min (0.17% relative error). Furthermore, we are aware that the external environmental conditions not being completely controlled had an impact on the performance of the sensors. It is still interesting to note the similar trends, regardless of the humidity. Statistical limitations are acknowledged due to the small sample size.

Further research on our part will involve more participants, in a room with a controlled environment, in order to test the system in various conditions to validate its robustness. Future work involving machine learning algorithms will need to be implemented in order to accurately predict breathing patterns, reduce the impact of noise, and detect breathing anomalies. It would also be interesting to take advantage of the presence of a number of sensors in the network to enable additional selectivity recognition as part of the known electronic nose approach. Sensors could then detect various components of breath, such as ketones and aldehydes [41,42,43].

## 4. Conclusions

This study demonstrates the integration of printed humidity sensors to detect breathing patterns and assess the humidity absorption of the mask’s membrane. The two 19-sensor arrays used in our study, completely covering the mask’s surface, detect variations in breathing patterns and model the sensors’ behavior over time, using an exponential decay model, with all $R^{2}>0.902$. Additionally, the sensors measure the respiration rate differences between deep and normal breathing across all participants, indicating sensitivity to the airflow. The generated heatmaps of airflow show great potential for further analysis in leak detection and mask fitting, as we can already detect differences in sensors’ behavior according to their position on the mask. Future work will focus on integrating machine learning models to personalize device performance and take into account the differences in breathing behavior amongst patients. We also plan to collect data for ill-fitted and well-fitted masks, to train a model that will detect air leaks for better user safety and improvement of the facemask’s protection. The findings of this study suggest that we can apply this system to half-face respirators and achieve similar results, which would represent a significant step forward in the development of intelligent protective equipment. Combining sensor arrays with machine learning will unlock the improvement of respiratory protection in workplaces and reduce exposure risks.

## Figures and Tables

**Table 1:** Ambient temperature and relative humidity for four participants across three days.

Participant	Day 1		Day 2		Day 3	
	Temp (°C)	R.H (%)	Temp (°C)	R.H (%)	Temp (°C)	R.H (%)
P1	23.7	21.4	22.9	51.3	22.7	51.3
P2	22.2	31.8	22.9	54.5	22.7	51.1
P3	23.9	21.2	22.3	55.4	23.0	50.2
P4	23.0	30.8	22.9	50.9	22.7	51.5
Mean	23.2	26.5	22.8	53.0	22.8	51.0

**Table 2:** $R^{2}$ coefficients of the exponential model fitting for all participants on all days.

	D1	D2	D3
P1	0.988	0.938	0.987
P2	0.984	0.976	0.902
P3	0.958	0.973	0.967
P4	0.988	0.982	0.907

**Table 3:** Respiration rate (RR) for all four participants on three consecutive days. Each wave (W) is an interval of 4 min normal breathing (N) + 1 min deep breathing (D). Average RR difference between both is included, as well as average signal amplitude difference.

Participant		W2		W3		W4		W5		W6		$\bar{\Delta \mathrm{RR}}$ (BPM)	$\bar{\Delta R}\;\left(\Omega \right)$
		N	D	N	D	N	D	N	D	N	D		
P1	D1	20.1	7.6	21.2	7.5	21.7	7.0	20.5	6.9	20.8	8.0	13.4	-
	D2	18.6	7.5	18.4	8.8	19.1	8.7	18.6	7.9	18.1	7.9	10.4	238.4
	D3	17.9	7.6	17.9	8.2	18.7	9.6	18.1	8.3	18.2	10.5	9.34	409.5
P2	D1	19.5	5.2	18.7	4.9	17.5	5.2	18.7	4.8	19.2	5.4	13.6	308.5
	D2	17.1	8.0	18.8	8.6	17.9	7.6	-	-	19.0	5.6	10.7	86.5
	D3	18.8	4.8	18.8	5.2	18.9	4.9	19.7	10.2	20.5	6.9	12.9	921.3
P3	D1	20.2	11.0	20.9	13.0	21.5	14.0	21.6	13.5	21.6	19.6	6.93	518.2
	D2	19.5	10.6	21.7	13.9	21.9	15.8	21.6	12.5	19.6	12.4	7.81	193.5
	D3	21.4	12.1	20.1	11.4	21.9	13.0	20.8	13.7	21.8	13.1	8.57	166.7
P4	D1	20.7	10.2	21.3	11.4	20.2	10.7	20.0	11.2	20.6	12.6	9.33	175.5
	D2	15.7	9.3	19.0	9.4	17.3	9.2	18.7	8.7	18.1	8.6	8.71	346.4
	D3	22.2	12.8	21.6	11.0	21.0	9.7	20.7	8.4	20.8	8.9	11.07	2805.8

## Data Availability

The dataset generated during and/or analyzed during the current study are available from the corresponding author on reasonable request.

## Supplementary Materials

The following supporting information can be downloaded at: https://www.mdpi.com/article/10.3390/s25247623/s1, Figure S1: Example of poor signal excluded for the computed mean; Figure S2: Modeling curves for all tests; Figure S3: Participant 1 breathing features for all tests; Figure S4: Participant 2 breathing features for all tests; Figure S5: Participant 3 breathing features for all tests; Figure S6: Participant 4 breathing features for all tests; Table S1: Excluded sensors for each test; Video S1: 3D animation of humidity variations in mask; Video S2: 2D animation of humidity variations in mask.
