# Supplementary material for: Assessment of KN95 Mask Filtering Degradation and Breathing Detection: A Pilot Study
Source: Sensors (Basel). 2025 Dec 16;25(24):7623. doi: 10.3390/s25247623 (PMC12736789; doi:10.3390/s25247623)
Supplement: Supplementary file 1 [file sensors-25-07623-s001.zip › sensors-3997619-supplementary.pdf]

Assessment of KN95 Mask Filtering Degradation and Breathing Detection: A Pilot Study Supplementary Information

Julie Payette[1], Alexandre Perrotton[1], Paul Fourmont[1,2], Fabrice Vaussenat[1], Jaime A. Benavides[1], Luis Felipe Gerlein[1], and Sylvain Cloutier[1,*]

[1]École de technologie supérieure, Department of Electrical Engineering, Montréal, H3C 1K3, Canada
[2]Department of Materials Science & Engineering, Massachussetts Institute of Technology, Cambridge, MA 02139, USA
[*]SylvainG.Cloutier@etsmtl.ca

ABSTRACT

This study aims to monitor mask performance *in operado* using all-printed humidity sensor arrays based on BiFeO3/BiOCl heterostructures. Two screen-printed 19-sensor arrays are fixed directly atop the mask, in order to analyze moisture levels in exhaled breath and extract performance indicators. This approach allows an examination of the humidity saturation and absorption over time during operation. Accumulation of moisture within the mask can affect its performances and factors like breath humidity, mask material and ambient conditions influence this. Results show that the measured data follows an exponential decay, achieving correlation factors over 0.9 for all tests. We also detect breathing differences through feature extraction, investigating the respiration rates and signal amplitudes for both normal and deep breathing. Furthermore, we animated the airflow in the mask in both 2D and 3D, allowing the eventual detection of leaks for ill-fitting masks. This study introduces an innovative approach for assessment of mask fit and longevity, contributing to improving mask efficacy and public health outcomes.

Detailed Methodology

Data collection

Table S1 how tells which sensors were excluded for each test. Figure S1 shows how the sensors' signal were skewed for certain time intervals. The peaks were excluded, as well as the time portion between 1000s and 1250s where the values were wrong. We excluded the least data possible, since the rest is suitable, as shown in the zoomed-in section.

Table S1. Excluded sensors for each test

Patient	Day 1	Day 2	Day 3
P1	-	S1-2	-
P2	S35,S37,S38		
P3	-	S1-7	S20
P4	-	-	S19

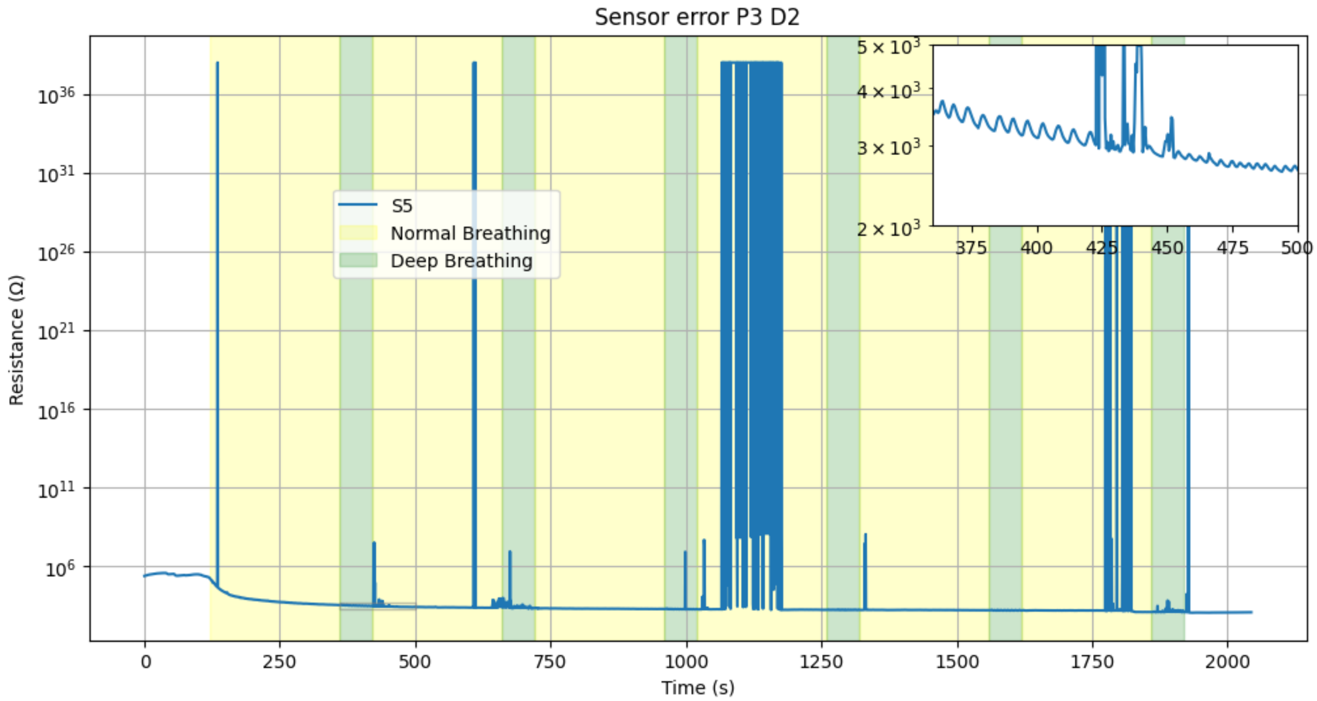

Figure S1. Example of poor signal excluded for the computed mean.

Supplementary Analysis

Model Fitting

Figure S2 shows all modeling curve for each test conducted.

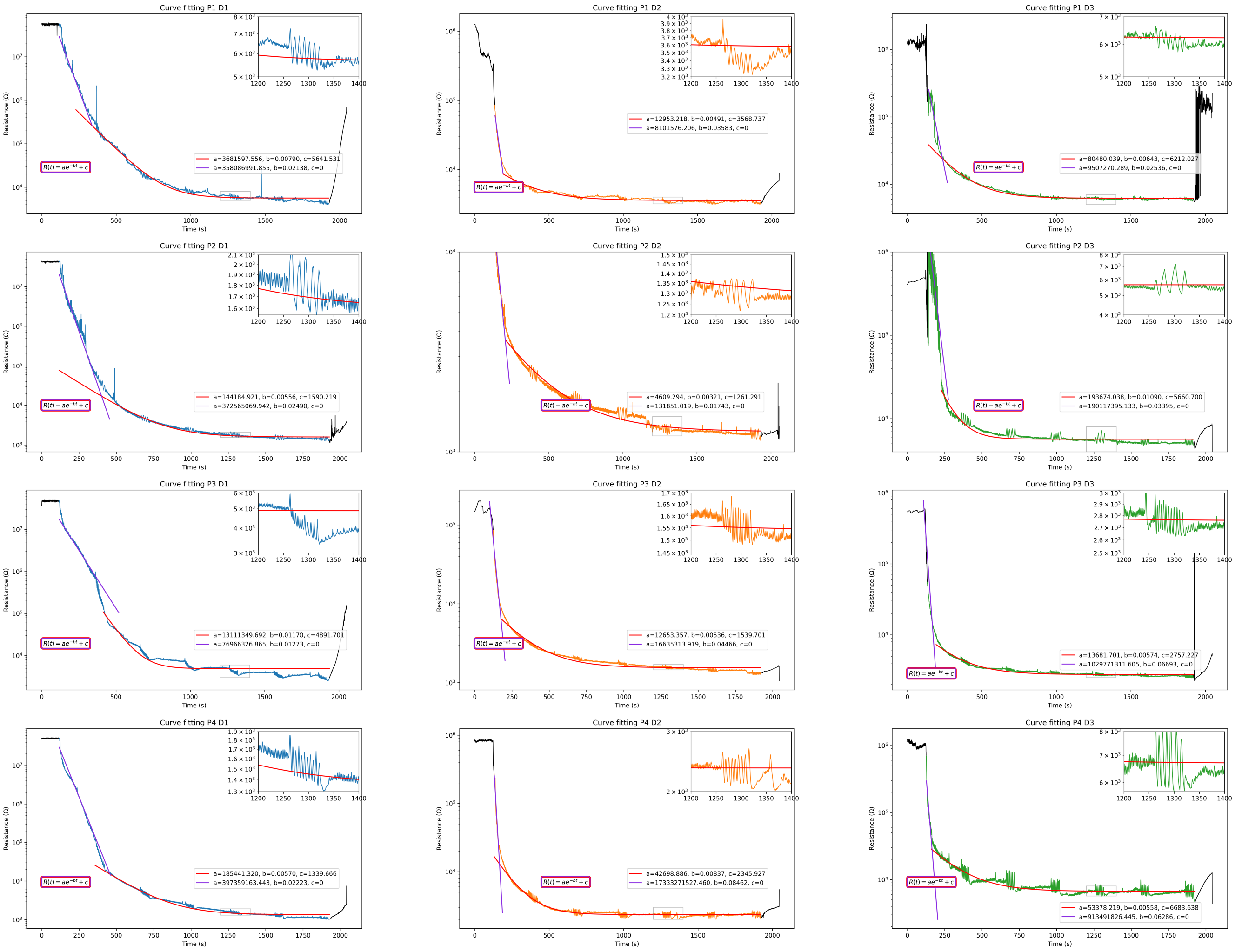

Figure S2. Modeling curves for all tests

Breathing Features

Both 2D and 3D animated videos of humidity variations can be viewed here : https://doi.org/10.5281/zenodo.17939536

Participant 1

Signal amplitude and breathing rate figures for Particpant 1. Day 1 amplitude was inconclusive, most likely due to the fact that the resistance on that test had a drastic decrease and a few outliers.

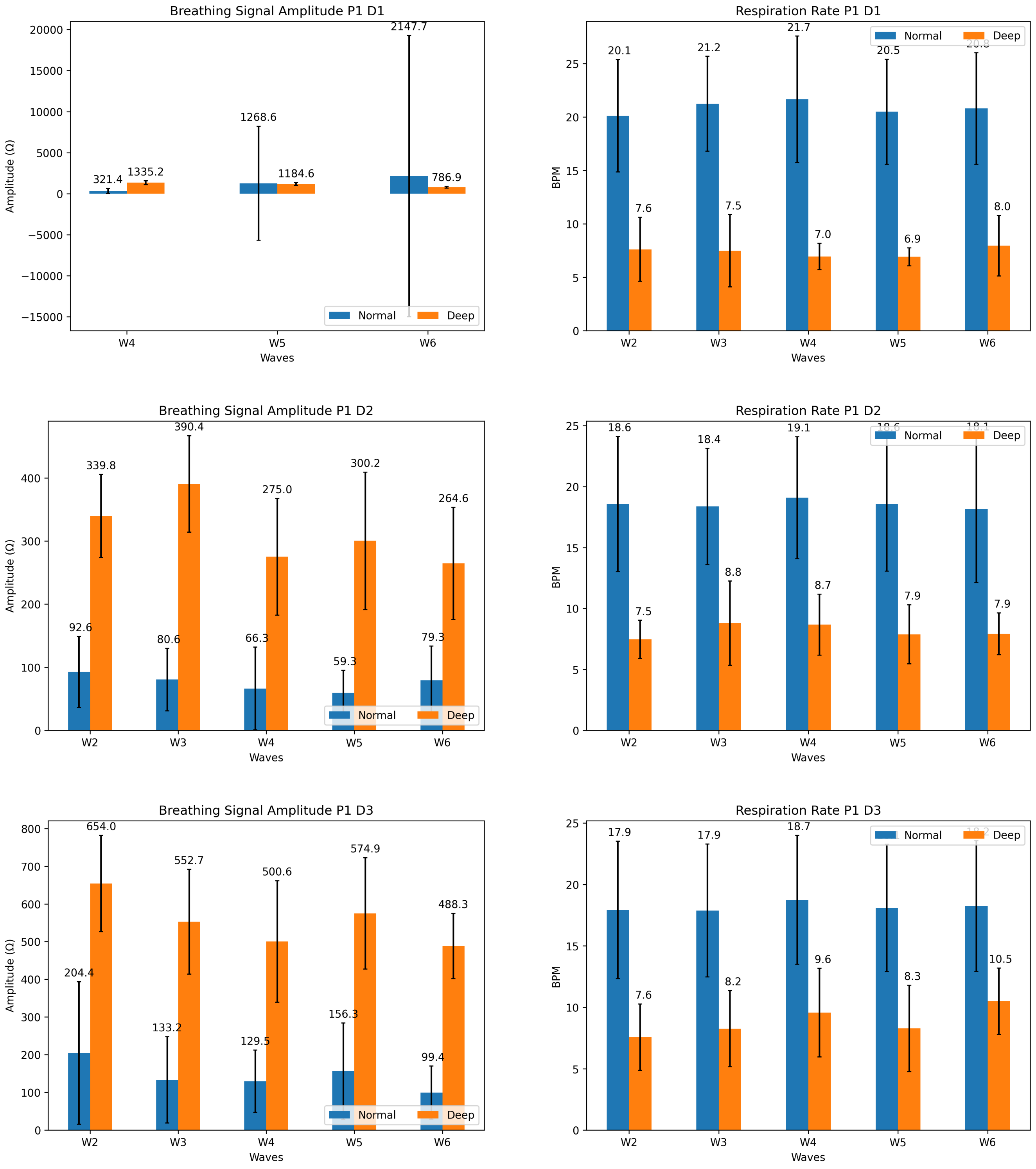

Figure S3. Participant 1 breathing features for all tests

Participant 2

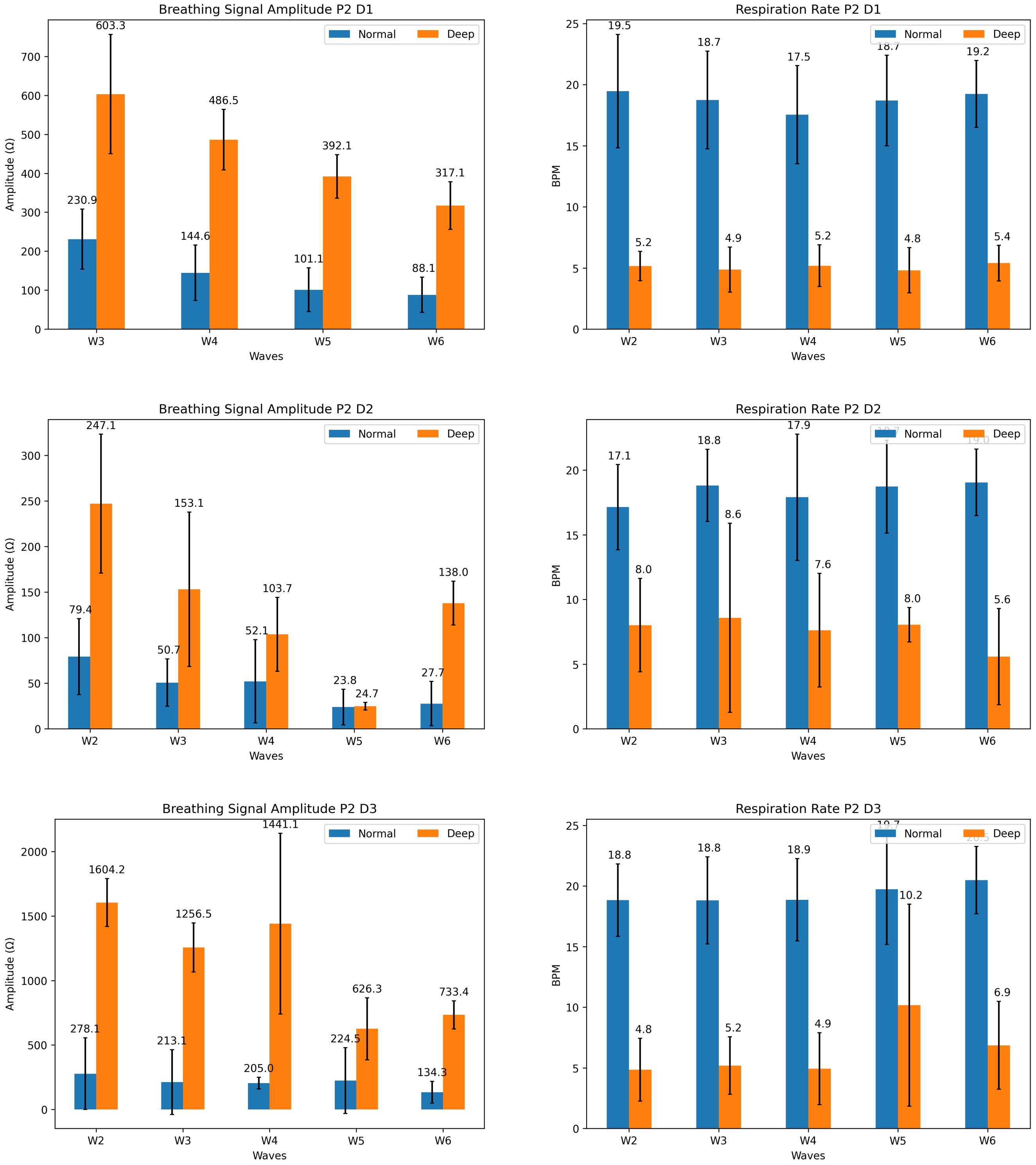

Figure S4. Participant 2 breathing features for all tests

Participant 3

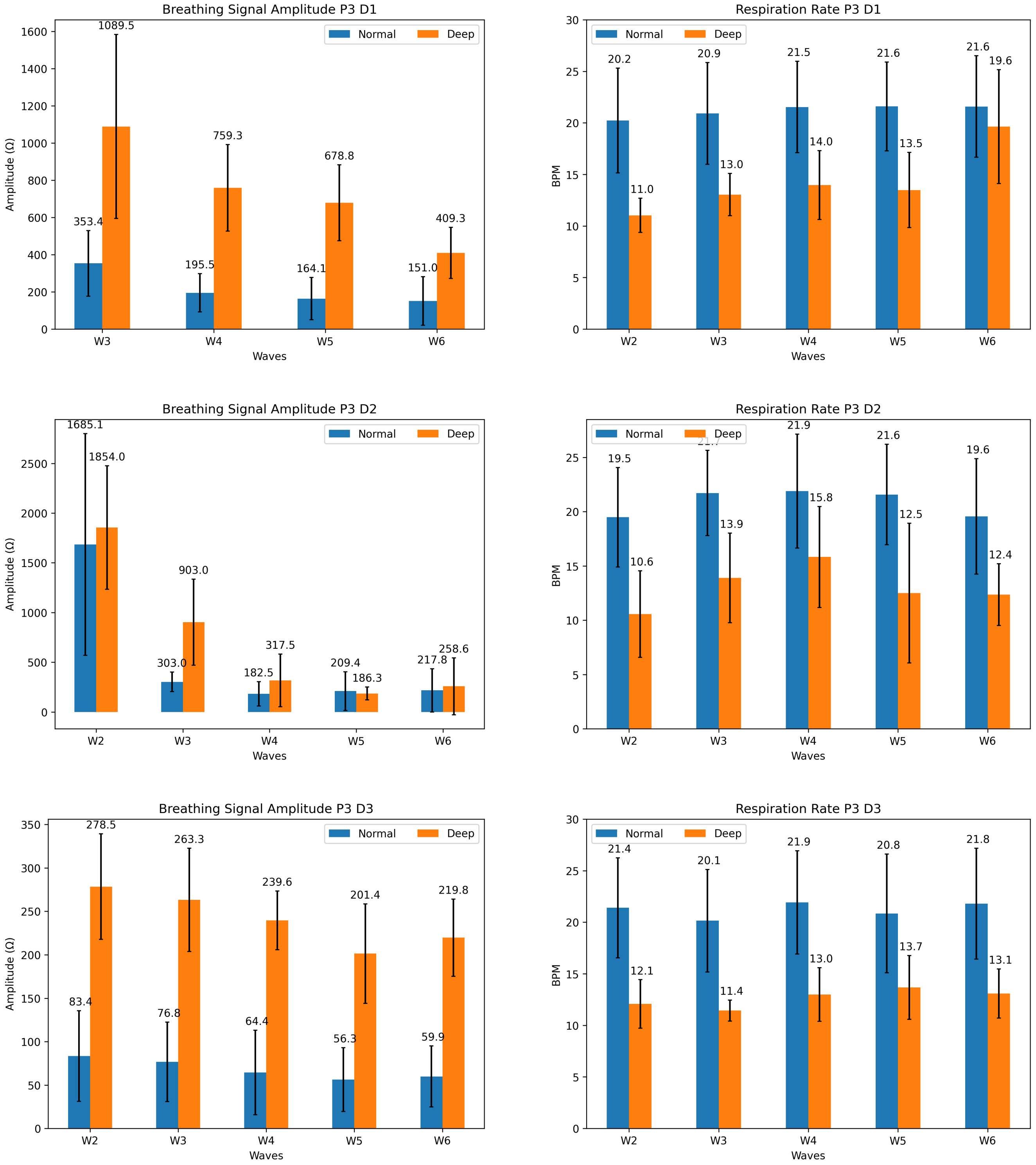

Figure S5. Participant 3 breathing features for all tests

Participant 4

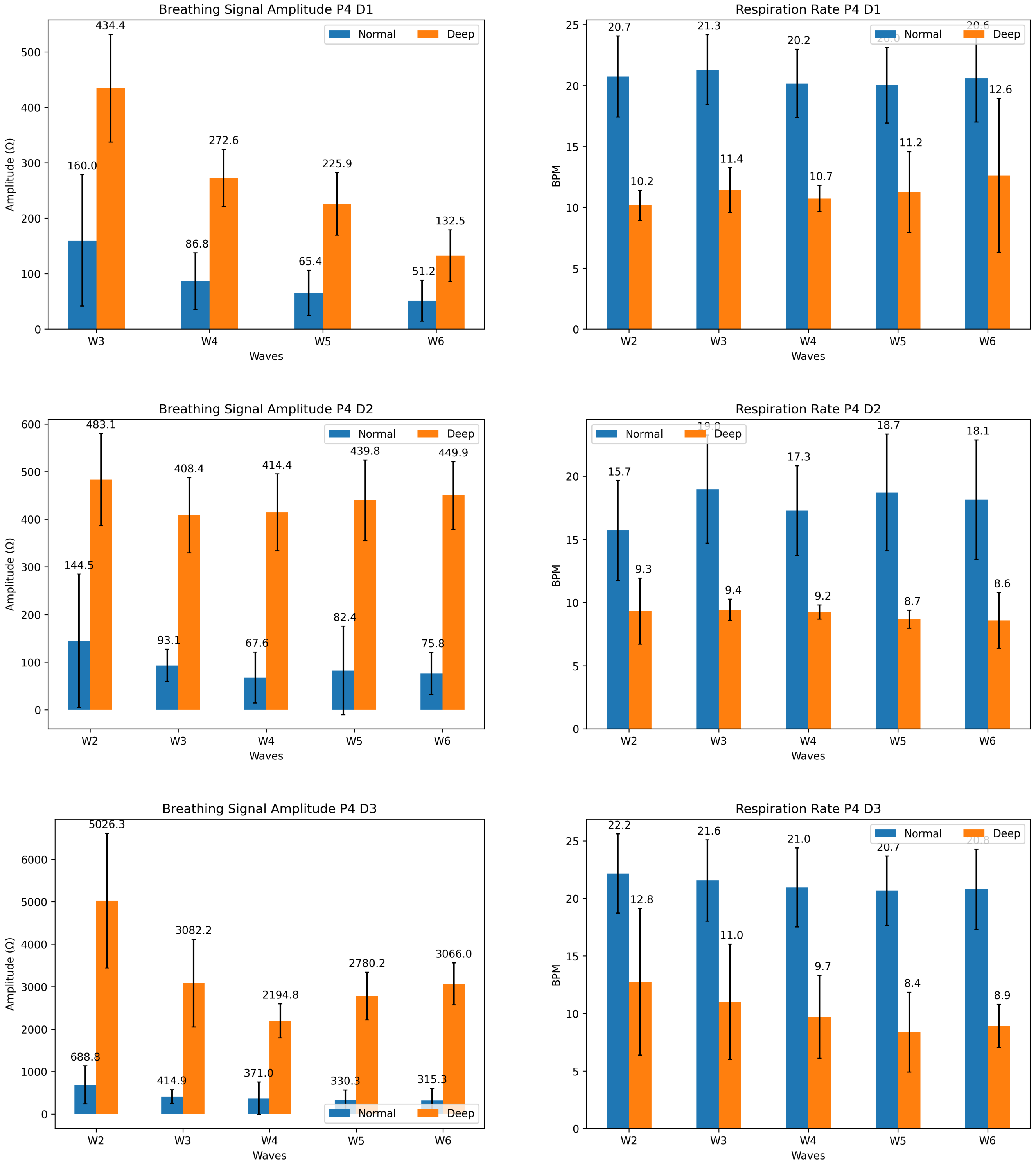

Figure S6. Participant 4 breathing features for all tests
